# *PTEN* homozygous deletion is a negative prognostic factor in tumor treating fields‐treated glioblastoma, IDH wildtype patients

**DOI:** 10.1111/bpa.70143

**Published:** 2026-09-10

**Authors:** Jakob Nückles, Claudius Jelgersma, Christin Siewert, Brendan Osberg, Simone Schmid, Arend Koch, Eilís Perez, Daniel Teichmann, Martin Misch, Anna‐Gila Karbe, Susanne Ukrow, Patrick N. Harter, Ruth M. Stassart, Peter Vajkoczy, Julia Onken, David Capper

**Affiliations:** ^1^ Department of Neuropathology Charité‐Universitätsmedizin Berlin, Corporate Member of Freie Universität Berlin and Humboldt Universität zu Berlin Berlin Germany; ^2^ Departement of Neurosurgery Charité‐Universitätsmedizin Berlin, Corporate Member of Freie Universität Berlin and Humboldt Universität zu Berlin Berlin Germany; ^3^ German Cancer Consortium (DKTK), Partner Site Berlin German Cancer Research Center (DKFZ) Heidelberg Germany; ^4^ Department of Pathology Unfallkrankenhaus Berlin (UKB) Berlin Germany; ^5^ Institute of Neuropathology, LMU Medizin Ludwig‐Maximilians‐Universität München Munich Germany; ^6^ German Cancer Consortium (DKTK), Partner Site Munich German Cancer Research Centre (DKFZ) Heidelberg Germany; ^7^ Bavarian Cancer Research Center (BZKF) Munich Germany; ^8^ Comprehensive Cancer Center (CCC) Munich University Hospital, Ludwig‐Maximilians‐University Munich Munich Germany; ^9^ Paul‐Flechsig‐Institut of Neuropathology University Clinic Leipzig Leipzig Germany

**Keywords:** glioblastoma, isocitrate dehydrogenase wildtype, overall survival, *PTEN* homozygous deletion, tumor treating fields

## Abstract

Tumor treating fields (TTFields) are a treatment for glioblastoma, isocitrate dehydrogenase wildtype (GBM, IDH wt) that improves survival, but no validated biomarkers exist to identify patients likely to benefit. This study aims to identify molecular biomarkers guiding clinical decisions on TTFields initiation. Eighty gliomas treated with surgery, radiochemotherapy, and TTFields were reassessed using DNA methylation profiling and TSO500 DNA/RNA panel sequencing, identifying 64 highly characteristic GBM, IDH wt with comprehensive molecular data. Alterations present in ≥5 cases were analyzed for association with survival using Kaplan–Meier and Cox models. Two TTFields‐naïve GBM, IDH wt cohorts (*n* = 175) served as controls. For 18 cases, post‐treatment tissue from a second surgery was profiled. Twenty‐four alterations occurred in ≥5 patients. Univariate analyses showed KPS and *MGMT* promoter methylation as protective, while *EGFR* amplification, *CDKN2A/B* loss, and homozygous *PTEN* deletion were linked to worse survival with TTFields. Multivariate analysis confirmed KPS and MGMT as protective and homozygous *PTEN* deletion as a risk factor (median OS 368 vs. 603 days; HR 3.86, *p* = 0.0049). In TTFields‐naïve cohorts, homozygous *PTEN* deletion was not associated with outcome, and frequencies were comparable (controls 7%, TTFields 11%). Post‐TTFields GBM, IDH wt did not acquire hypermutation or other recurrent molecular alterations. Alongside the established factors *MGMT* and KPS, *PTEN* homozygous deletion was associated with worse survival in GBM, IDH wt patients treated with TTFields. Homozygous *PTEN* deletion may be associated with reduced benefit from TTFields and should be further investigated as a predictive biomarker for TTFields treatment.

## Graphical abstract

In a molecularly confirmed cohort of 64 newly diagnosed, TTFields‐treated glioblastomas, IDH‐wildtype, homozygous *PTEN* deletion was associated with markedly shorter overall survival (368 vs. 603 days) and remained an independent adverse prognostic factor. These findings warrant prospective validation of *PTEN* homozygous deletion as an adverse biomarker in TTFields‐treated glioblastoma, IDH‐wildtype.

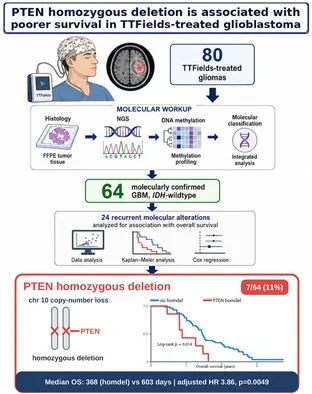

## 1 INTRODUCTION

Glioblastoma, isocitrate dehydrogenase wildtype (GBM, IDH wt), is the most common and aggressive primary brain tumor in adults. Despite maximal standard‐of‐care treatment with surgery, radiotherapy, and concomitant plus adjuvant temozolomide, median overall survival remains only 15–18 months [1]. Over the past decade, tumor treating fields (TTFields) have been introduced as an additional treatment modality. By applying low‐intensity, intermediate‐frequency alternating electric fields (100–500 kHz), TTFields have been demonstrated to disrupt mitotic spindle assembly and cytokinesis, thereby inhibiting tumor cell proliferation [2, 3]. Preclinical studies have implicated diverse molecular consequences of TTFields application, ranging from microtubule disruption and activation of the *PI3K/AKT/mTOR* pathway to changes in gene expression, transcriptomics, and proteomics [3, 4, 5]. However, it remains unclear whether TTFields induce specific genomic or epigenomic alterations in human tumors, and to what extent molecular features present in the tumor may influence clinical benefit. Furthermore, it is increasingly recognized that TTFields are gaining importance in clinical practice, extending beyond glioblastoma to include other cancers such as lung, pancreatic, and mesothelioma, as well as additional WHO grade 4 gliomas [6, 7, 8, 9]. For GBM, IDH wt the question remains which patients may benefit most from TTFields therapy and which may actually benefit less. Toms et al. demonstrated in a subgroup analysis of the EF‐14 Phase III trial that TTFields device usage is independently prognostic for survival in newly diagnosed glioblastoma [10]. To ensure adequate treatment compliance with the TTFields device, therapy is often prescribed to patients with a high Karnofsky Performance Status (KPS), in accordance with the inclusion criterion of KPS ≥70 in the pivotal EF‐14 trial [11]. This is reflected not only by the relatively high KPS scores reported in clinical trials, but also by the requirement of major insurers such as Medicare/CMS in the United States, which mandate a KPS ≥70 for reimbursement [12]. Beyond efficacy, real‐world use of TTFields hinges on acceptance and sustained device usage; continuous wear can interfere with daily life and quality of life and entails time and socioeconomic costs. Accordingly, biomarker‐guided selection could both avoid overtreating patients unlikely to benefit and, conversely, enable motivated patients with functional limitations to receive TTFields when molecular profiles are favorable. However, the question to what extent molecular features of GBM, IDH wt may influence the clinical benefit of TTFields remains open. Since the 5th edition of the WHO Classification of Tumors of the Central Nervous System (2021), the modern classification of brain tumors increasingly relies on molecular characteristics [13]. Consequently, analytical techniques such as next‐generation sequencing (NGS) of DNA and RNA, as well as genome‐wide DNA methylation profiling, are commonly used to capture disease‐defining molecular features. Using DNA methylation profiling, targeted NGS, and copy number analysis, we aimed to identify molecular biomarkers associated with TTFields treatment outcomes. To address these questions, we conducted an integrated clinical and molecular analysis of a well‐defined, molecularly confirmed cohort of newly diagnosed GBM, IDH wt patients treated with first line TTFields at our institution.

## 2 MATERIALS AND METHODS

### 2.1 Clinical data

Clinical data were retrospectively collected from medical records at the Department of Neurosurgery Charité‐Universitätsmedizin Berlin, including demographics, preoperative Karnofsky performance status (KPS), pharmacological treatment, and disease progression data. Survival data were last updated May 2023. The main endpoint was overall survival (OS), defined as time from primary surgery to death; living patients were censored at last follow‐up. Extent of resection (EoR) was determined by 48‐h post‐op MRI and categorized as gross total resection (GTR: complete removal), subtotal resection (STR: >90% removal), or partial resection/biopsy (<90%) of the contrast enhancing tumor parts. For TTFields, data on treatment duration and device usage were recorded on a daily basis and extracted from the monthly usage reports. Therapy duration was calculated from start to end dates.

### 2.2 Tissue preparation

For genetic and epigenetic analyses, HE sections of formalin‐fixed, paraffin‐embedded (FFPE) tissue were assessed by a board‐certified neuropathologist (D.C.), and regions with an estimated tumor cell content (TCC) of ~80% were selected. In recurrent tumors with lower TCC, the area with highest TCC was chosen. Tissue was excised from marked regions using a 1‐mm tissue punch (depth ≤3 mm). After deparaffinization, DNA and RNA were extracted using the RSC FFPE Plus DNA/RNA Kits (Promega, Fitchburg, WI) and Maxwell DNA Extractor, then diluted in 40 μL ddH_2_O. Concentrations were determined fluorometrically: DNA with Quantus™ Fluorometer (Promega), RNA with Qubit 4 Fluorometer (Thermo Fisher Scientific, Waltham, MA).

### 2.3 Targeted next generation sequencing using the TruSight™ Oncology 500 Assay

For comprehensive genetic profiling, the TruSight™ Oncology 500 Assay (Illumina, San Diego, CA) was used to detect 523 cancer‐related genes and 55 structural variants, including gene fusions and splice variants from RNA. The panel also enables calculation of tumor mutational burden (TMB). RNA was denatured, primed with random hexamers, and reverse‐transcribed into cDNA. DNA and RNA underwent end repair, A‐tailing, and adapter ligation. Library preparation required ≥40 ng DNA and ≥80 ng RNA with RV200% >20%. Enrichment was done via two‐step hybridization and capture, followed by PCR amplification. Quality control criteria included library concentration ≥3 ng/μL, median insert size ≥70 bp, and ≥90% of exons covered at ≥50×. Libraries were pooled and sequenced on a NextSeq 550 system (Illumina, San Diego, CA).

### 2.4 Selection and assessment of biologically relevant genetic variants from panel‐based NGS results

NGS mutation raw data were processed using TSO500 Local App v2.2.0 or DRAGEN TSO 500 Analysis Software v2.5.2.1 (Illumina, San Diego, CA), aligned to the GRCh37 (hg19) reference genome. Quality control included mean target coverage (≥500) and contamination assessment per manufacturer's guidelines. Germline variants were predicted via the DRAGEN Germline Small Variant Caller, but only somatic variants were included in downstream analysis. Variant calling for SNVs and indels was performed using Pisces v5.2.10 (Illumina, San Diego, CA), and annotation was conducted with ANNOVAR. Gene variants were selected based on coding impact, including: *missense_variant*, *stop_gained*, *splice_region_variant*, *inframe_deletion*, *frameshift_variant*, *inframe_insertion*, *splice_acceptor_variant*, *splice_donor_variant*, and *start_lost*. Only variants classified as “*likely pathogenic*” or “*pathogenic*” according to ACMG criteria were considered, with variant classification performed using the TAPES algorithm (Tool for Assessment and Prioritisation in Exome Studies) [14, 15]. Additionally, TP53 mutations not meeting ACMG pathogenicity but identified as known hotspots were included [16, 17]. *TERT* promoter mutations (c.‐124C>T [G228A] and c.‐146C>T [G250A]) were classified as pathogenic regardless of ACMG status, in line with 2021 WHO diagnostic criteria for GBM, IDH wt. A minimum variant allele frequency (VAF) of 5% was required. Final variant calls were visually verified using Integrative Genomics Viewer (IGV).

### 2.5 RNA data analysis

For detection of structural gene alteration such as gene fusions and splice variants, NGS RNA raw data were aligned to hs37d5 with STAR v2.7.11a (GENCODE19; ‐‐chimSegmentMin 10 ‐‐chimJunctionOverhangMin 10 ‐‐chimScoreMin 1) and sorted/indexed with SAMtools [18, 19, 20, 21]. Arriba v2.4.0 called and visualized gene fusions (blacklist filtering, COSMIC annotation; cytobands/protein domains), and splice variants were identified with the DRAGEN TSO500 pipeline (v2.6.0.2) [22].

### 2.6 Calculation of tumor mutational burden

Tumor mutational burden (TMB) was calculated using the Illumina algorithm (pipeline ruo‐2.2.0.12), based on somatic synonymous and non‐synonymous variants with ≥50× coverage and allele frequency ≥5%. The coding target region size used for TMB estimation was 1.28 Mb, per manufacturer specifications.

### 2.7 Methylation analysis

DNA samples (≥250 ng) were bisulfite‐converted using the EpiTect Fast DNA Bisulfite Kit (QIAGEN, Netherlands) to convert unmethylated cytosines to uracil while preserving methylated cytosines. DNA restoration was performed with the Infinium™ HD FFPE Restore Kit (Illumina, San Diego, CA), followed by hybridization to Infinium MethylationEPIC BeadChips (850 K v1.0 or 935 K v2.0). Arrays were scanned using the iScan system (Illumina, San Diego, CA) for high‐resolution profiling. Raw IDAT files underwent quality control and were uploaded to molecularneuropathology.org/mnp. Classification used the brain classifier v12.8, trained on 7495 methylation profiles, expanding the taxonomy from 91 classes in v11 to 184 subclasses in v12.8 [23, 24]. As previously suggested, we defined that a classifier score ≥0.84 indicated a match with a methylation class. Subclass assignment (e.g., receptor tyrosine kinase 1) was performed for cases that reached classifier score ≥0.84 for the class and additionally reached a cutoff of ≥0.5 for subclass assignment.

### 2.8 Determination of Copy number variations and chromosomal aberrations

Copy number variations (CNV) plots were derived from methylation data using the R package *conumee* v1.30.0 (Bioconductor). Each plot was manually assessed considering both histologically determined and visually estimated TCC, as low TCC can cause flattened chromosomal plots. Because of variability in TCC and inter‐array performance, no fixed log2 cutoff was applied; instead, the following specific visual and contextual criteria were used.

### 2.9 Combined +7/−10 chromosomal aberration

Defined as present when >50% of chromosomes 7 and 10 showed deviation from baseline, indicating trisomy 7 and monosomy 10 (Figure S1C, Supporting Information).

### 2.10 Gene amplifications

A gene was classified as amplified when a focal elevation of the baseline (blue) accompanied by a distinct row of green dots spanning the gene locus was observed, positioning the gene clearly outside the surrounding green cloud (Figure S1B). All amplified genes showed log2 values ≥0.6. Evaluated genes: *EGFR*, *PGFRA*, *FGFR*, *CDK4*, *CDK6*, *MDM4*, *MDM2*, *MYC*, *MYCN*, *MET*, *CCND2*, *TERT*.

### 2.11 Gene deletions and loss of heterozygosity

Deletions were classified as balanced, heterozygous, or homozygous. Chromosome 10 loss in GBM IDH wt served as reference for heterozygous loss (Figure S1). A gene was considered heterozygously deleted if its signal matched that of chr10 loss. Homozygous deletions (e.g., *PTEN*) were defined as deviations ≥2× the chr10 loss distance (Figure S1C). One case without chr10 loss was defined as homozygous *CDKN2A/B* deletion based on a −log2 value of −1.3. Genes with no or minimal deviation from baseline were classified as balanced. The following genes were evaluated: *PTEN*, *CDKN2A/B*, *RB1*, *NF1*, *TP53*. For selected cases harboring *PTEN* mutations without concurrent chromosome 10 deletion, loss of heterozygosity (LOH) was assessed using PureCN [25] applied to TruSight Oncology 500 (TSO500) targeted sequencing data, with CNVkit‐derived copy number segments and GATK Mutect2 somatic variant calls as input. LOH status was determined by visual inspection of allele‐specific copy number profiles and B‐allele frequency patterns in the PureCN output.

### 2.12 MGMT promoter methylation

*MGMT* promoter methylation status was assessed in 59 cases using pyrosequencing. In an additional subset of 5 cases with methylation array data, status was binarily classified as methylated or unmethylated using the *MGMT*‐STP27 model based on CpG sites cg12434587 and cg12981137 [26].

### 2.13 *PTEN* immunohistochemistry

*PTEN* immunohistochemistry (IHC) was performed on available FFPE tumor tissue using a rabbit monoclonal anti‐*PTEN* antibody (clone 138G6, Cell Signaling Technology, catalog no. 9559; rabbit IgG). Only cases with evaluable tumor tissue and unequivocal internal positive control staining were included. In line with previous glioma studies [27, 28], *PTEN* expression in tumor cells was assessed semi‐quantitatively using a three‐tier scoring system: 0, complete loss of *PTEN* expression; 1, weak or reduced *PTEN* expression; and 2, retained *PTEN* expression. For outcome analyses, *PTEN* IHC scores were analyzed both as three groups (score 0, 1, and 2) and as a binary variable comparing complete *PTEN* loss (score 0) with weak/retained expression (scores 1 or 2).

### 2.14 Statistical analysis

Molecular alterations present in ≥5 patients were analyzed for associations with OS using Kaplan–Meier estimates and univariate as well as multivariate Cox models. In addition to molecular alterations, baseline clinical variables including age at primary resection, sex, preoperative Karnofsky performance status (KPS), and extent of resection were evaluated for association with OS. TTFields treatment duration and average TTFields usage rate were recorded and reported descriptively but were not included as baseline covariates in the primary survival models. Treatment duration is inherently dependent on survival time and may therefore introduce immortal time bias. Average TTFields usage rate, although clinically relevant as an adherence‐related variable, is measured after treatment initiation and may be influenced by clinical deterioration, early progression, tolerability, performance status, and patient‐related factors. It is therefore susceptible to selection bias and reverse causation. Accordingly, both TTFields‐related variables were interpreted cautiously and were not used for biomarker selection or inclusion in the multivariate model. Only alterations significant in both univariate Kaplan–Meier and univariate Cox analyses were included in the multivariate model. For continuous variables such as KPS and age, statistical significance in the univariate Cox model was considered sufficient for inclusion in the multivariate model. The proportional hazards assumption was tested for all Cox models; no individual or global *p*‐value was allowed to fall below 0.05. To assess independence among variables (e.g., *MGMT*, KPS), multicollinearity was evaluated using a linear model with a dummy outcome variable. Variance inflation factors (VIFs) were <2 for all variables, indicating low multicollinearity. *PTEN* IHC scores were analyzed for association with OS using Kaplan–Meier estimates and log‐rank tests, *PTEN* loss (score 0) with weak or retained *PTEN* expression (scores 1 or 2). As an exploratory analysis, putative biallelic *PTEN* inactivation was analyzed using Kaplan–Meier estimates and a log‐rank test. Putative biallelic *PTEN* inactivation was defined as either homozygous *PTEN* deletion or a pathogenic *PTEN* mutation accompanied by concurrent heterozygous *PTEN* deletion.

### 2.15 Controls

Two TTFields‐naïve cohorts with newly diagnosed GBM, IDH wt (combined *n* = 175) served as controls. TCGA samples were obtained via TCGA Biolinks [29]; molecular and clinical data for the Lucas et al. cohort were extracted from the original publication [30]. Clinical TCGA‐GBM data were supplemented using the MC3 MAF file [31]. Diagnoses were confirmed using the provided DNA methylation data, and *PTEN* deletion status was assessed from copy number plots as described above.

## 3 RESULTS

### 3.1 Patient selection and molecular confirmation of diagnosis

**FIGURE 1:**
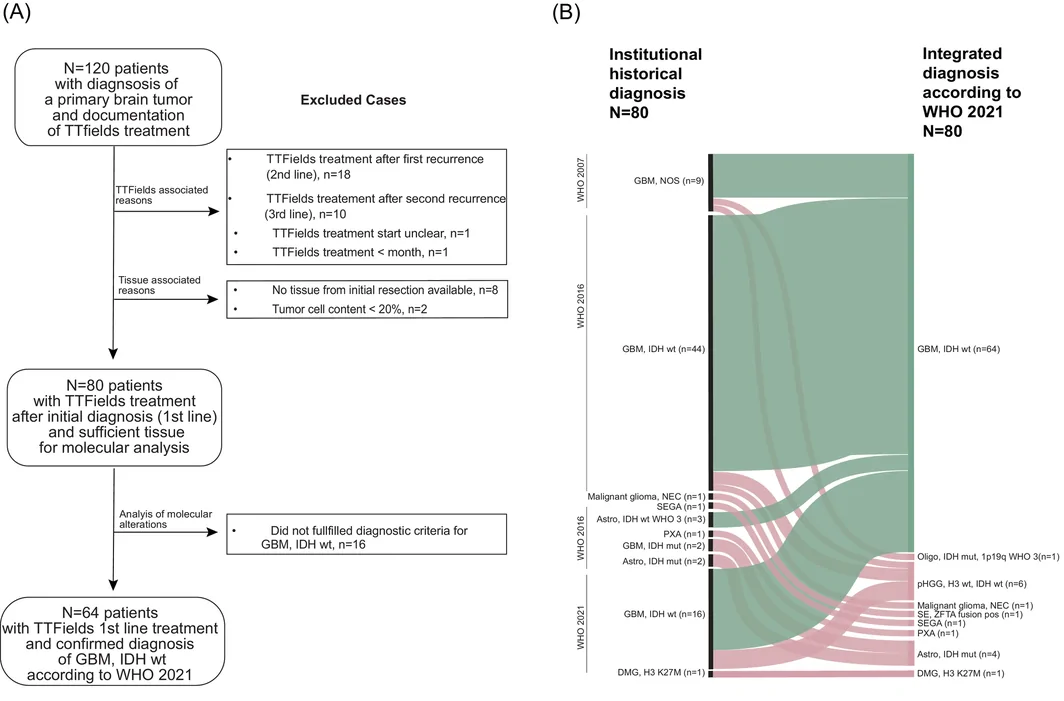
Patient selection process and results of molecular classification. (A) Inclusion and exclusion algorithm of patients with documented TTFields treatment at the Department of Neurosurgery Charité‐Universitätsmedizin Berlin. The rigorous exclusion resulted in a highly homogenous cohort of 64 molecularly confirmed Glioblastomas, IDH wt with well documented TTF treatment data. (B) Sankey plot depicting the exclusion of tumors not representing glioblastoma, IDH wt by DNA methylation profiling. From the 80 tumors screened, 64 were confirmed as definite glioblastomas, IDH wildtype and were further considered for the study. Astro = astrocytoma; DMG, H3 K27M = diffuse midline glioma, H3 K27M; NEC = not elsewhere classified; NOS = not otherwise specified; mut = mutant; OLIGO = oligodendroglioma; pHGG, H3 wt, IDH wt = pediatric high‐grade glioma, H3 wt, IDH wt; PXA = pleomorphic xanthoastrocytoma; SEGA = subependymal giant cell astrocytoma; SE, ZFTA fusion‐positive = supratentorial ependymoma, ZFTA fusion‐positive; wt = wildtype.

Initially, 120 patients with high‐grade gliomas treated with TTFields between June 2015 and Mai 2023 were identified from the Department of Neurosurgery's institutional database at Charité Universitätsmedizin Berlin. A graphical overview of patient inclusion and exclusion is provided in Figure 1A. To ensure a uniform treatment protocol, we included only first‐line TTFields patients treated after initial diagnosis thereby excluding 29 patients from the initial cohort. Another patient was excluded because the treatment duration was less than 1 month. Additionally, 8 patients were excluded because of the unavailability of tissue from the initial resection, and 2 because their resected tissue contained too little tumor cell content for molecular analysis. To achieve a molecularly homogeneous cohort, the remaining 80 tumors underwent DNA methylation profiling and NGS to verify all diagnoses at the molecular level. Sixty‐four cases were classified as GBM, IDH wt, whereas 16 were found to have other diagnoses and were excluded. These were 4 IDH mut astrocytomas, 6 diffuse pediatric‐type high‐grade gliomas, H3‐wildtype and IDH wt, and other rare tumor classes. Figure 1B depicts the final classification of the 80 high‐grade gliomas analyzed by DNA methylation profiling with 64 molecularly confirmed GBM, IDH wt.

### 3.2 Patient characteristics

Clinical characteristics of the final TTFields cohort (*n* = 64) can be found in Table 1. Notably, the median age in our cohort was relatively young, at 55.5 years [range 39–80], possibly reflecting the greater willingness of younger patients to consider additional therapeutic options. There was also a sex imbalance (62.5% men vs. 37.5% women), consistent with the known male predominance in GBM, IDH wt. The cohort was clinically homogeneous with respect to preoperative KPS, EoR, and clinical treatments administered alongside TTFields therapy, reflecting an exceptionally uniform GBM, IDH wt cohort. Most patients (82.8%) exhibited a preoperative KPS score greater than 80. Regarding EoR, at least STR or GTR was achieved in 76.5% of patients. Importantly, 90.6% of patients received standard treatment according to the Stupp protocol [1]. TTFields‐specific variables were consistent with those reported in other study cohorts, showing a median usage rate of 67% [range 21–93%] throughout the treatment duration and a median treatment duration of 5.93 months [range 1–35.39 months] [32, 33, 34]. Clinical variables including age at primary resection, sex, preoperative KPS, and extent of resection were analyzed for association with OS. The results of univariate cox regression are presented in Table S2.

**TABLE 1:** Patient and treatment characteristics of the first‐line TTFields‐treated newly diagnosed glioblastoma cohort (IDH‐wildtype; WHO 2021), *n* = 64.

Characteristics	
Sex, *n* (%)	
Male	40 (62.5)
Female	24 (37.5)
Age at primary resection [year] median (range)	55.5 (39–80)
Preoperative KPS, *n* (%)	
100	3 (4.7)
90	34 (53.1)
80	16 (25)
<70	7 (10.9)
NA	3 (4.7)
Extension of resection, *n* (%)	
GTR	34 (53.1)
STR	15 (23.4)
Partial resection or biopsy	10 (15.6)
NA	5 (7.8)
Tumor localization, *n* (%)	
Frontal lobe	17 (26.6)
Parietal lobe	16 (25.0)
Temporal lobe	21 (32.8)
Occipital lobe	7 (10.9)
Multifocal	3 (4.7)
Clinical treatment in addition to TTFields treatment	
RTX + TMZ	59 (90.6)
RTX + TMZ + CCNU	2 (3.1)
Best supportive care	1 (1.5)
NA	1 (1.5)
Re‐resection, *n* (%)	
Yes	20 (31.3)
No	43 (67.2)
TTFields start cycle of TMZ, *n* (%)	
TMZ #1	19 (30.0)
TMZ #2	11 (17.2)
TMZ #3	12 (18.8)
TMZ #4	7 (10.9)
TMZ #5	4 (6.3)
After TMZ#6	7 (10.9)
NA	4 (6.3)
Usage of TTFields [%/day]	
Average	66
Median [range]	67 [21–93]
Therapy duration [months]	
Average	7.96
Median [range]	5.93 [1–35.39]

*Note*: Data are reported as *n* (%) unless otherwise indicated (median [range] or mean).

Abbreviations: CCNU, lomustine; GTR, gross total resection; KPS, Karnofsky performance status; RTX, radiotherapy; STR, subtotal resection; TMZ, temozolomide.

### 3.3 Characteristics of molecularly confirmed GBM, IDH wt TTFields cohort

**FIGURE 2:**
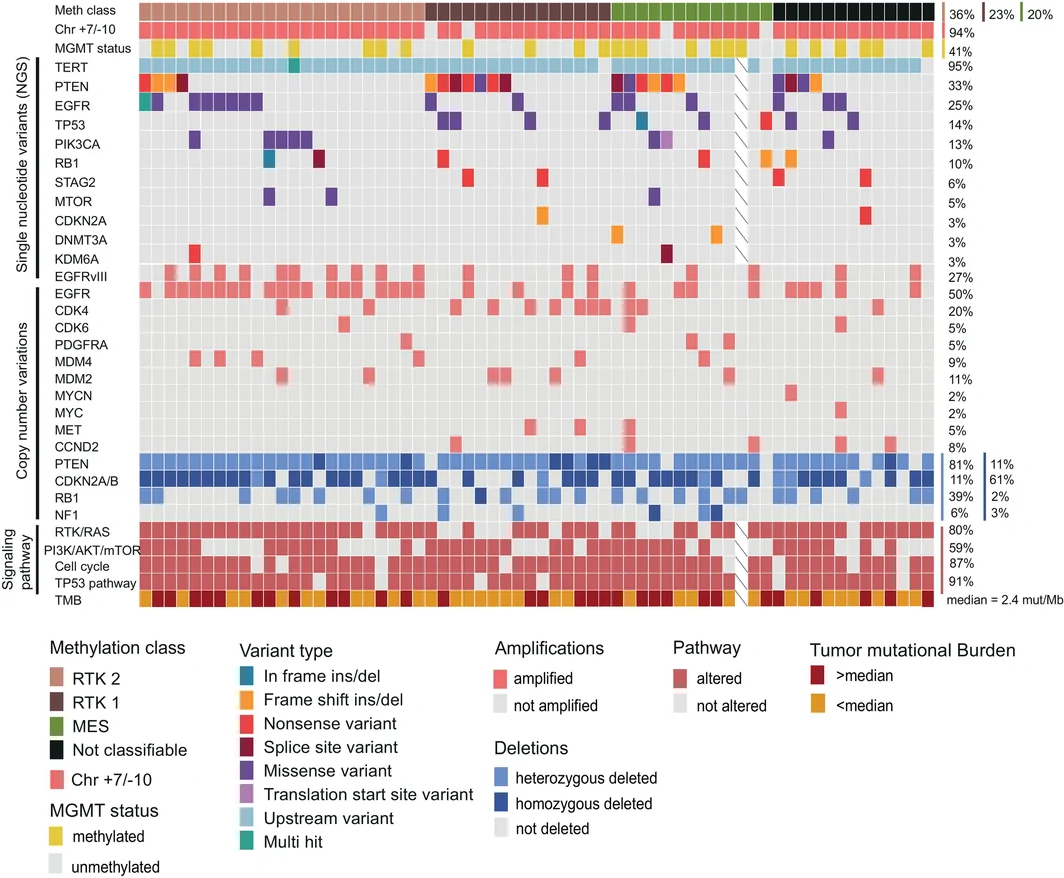
Oncoplot summarizing the molecular landscape of *N* = 64 patients with newly diagnosed GBM, IDH wt (WHO 2021), treated with TTFields. Shown are the most frequent genetic alterations detected by next‐generation sequencing, DNA methylation profiling and copy number analysis. The differently colored tiles within a vertical column represent the molecular alterations of an individual patient. A grey tile indicates the absence of an alteration. At the top, diagnostically relevant molecular criteria are shown, including methylation class (shades of brown, green and black), chromosome +7/−10 aberration (in red), *MGMT* promoter methylation status (yellow), and *TERT* promoter mutation (light blue). On the left, cancer‐relevant genes (e.g., *PTEN*, *EGFR*, *TP53*) are listed, with the respective mutation types indicated by the color legend. Gene deletions (shades of blue) and amplifications (lighter red) derived from copy number profiles are depicted in the lower block. The *EGFRvIII* splice variant is also shown in light red. In addition, genetic pathway activation affecting the most frequently altered signaling pathways in GBM, IDH wt are shown in darker red. For tumor mutational burden (TMB), the cohort was dichotomized at the median of 2.4 mut/Mb, with cases below the median shown in orange and cases above the median shown in dark red. On the right, the relative frequencies of all molecular alterations are indicated as percentages. For one case NGS data was not available (crossed fields).

Detailed molecular results of the study cohort of 64 molecularly confirmed GBM, IDH wt are provided as an Oncoplot in Figure 2. DNA methylation subclass classification revealed 36% (*N* = 23/64) receptor tyrosine kinase 2 (*RTK2*) GBM, IDH wt subclass, 23% (*N* = 15/64) receptor tyrosine kinase 1 (*RTK1*), 20% (*N* = 13/64) mesenchymal (*MES*), and 20% (*N* = 13/64) of cases where the methylation subclass could not be assigned unambiguously. Disease‐defining features such as the *TERT* promoter mutation and +7/−10 chromosomal aberration were identified in 95% and 94% of cases, respectively, underscoring the high molecular uniformity of the study cohort. *MGMT* promoter methylation status showed a higher proportion of patients with unmethylated (59%) compared to methylated (41%) promoters. The most frequent mutations identified by DNA sequencing involved *PTEN* (33%, *N* = 21/63), *EGFR* (25%, *N* = 16/63), *TP53* (14%, *N* = 9/63), *PIK3CA* (13%, *N* = 8/63), and *RB1* (10%, *N* = 6/63) genes. Notably, no *NF1* mutations were detected, reflecting a somewhat lower overall incidence than expected for GBM, IDH wt. Frequent focal high‐level amplifications involved *EGFR* (50%, *N* = 32/64), *CDK4* (20%, *N* = 12/64), and *MDM2* (11%, *N* = 7/64). As expected, the *EGFRvIII* splice variant identified in RNA sequencing consistently co‐occurred with *EGFR* amplification (27%, *N* = 17/64). Homozygous deletions primarily affected tumor suppressor genes such as *CDKN2A/B* (61%; *N* = 39/64), *PTEN* (11%; *N* = 7/64), *NF1* (3%, *N* = 2/64), and *RB1* (2%, *N* = 1/64). Heterozygous deletions were also observed, most frequently in *PTEN* (81%; *N* = 52/64), *RB1* (39%, *N* = 25/64), *CDKN2A/B* (11%; *N* = 7/64), and *NF1* (6%, *N* = 4/64). Two cases in the series showed *PTEN* mutations by NGS but did not exhibit the prototypical chromosome 10 loss. We therefore performed loss‐of‐heterozygosity analysis using the NGS data to investigate the status of the second allele. Case 1 showed B‐allele frequency changes suggestive of copy‐neutral loss of heterozygosity (LOH) on chromosome 10, indicating that the *PTEN* wild‐type allele was likely lost in the tumor. In contrast, case 2 showed no convincing evidence of chromosome 10 LOH. The latter instead displayed segmental allelic imbalances in the context of a highly aneuploid/polyploid genome. Importantly, both *PTEN*‐mutated cases without concurrent chromosome 10 loss showed complete loss of *PTEN* expression by immunohistochemistry (score 0), indicating loss of detectable *PTEN* protein expression in both tumors despite the absence of broad chromosome 10 loss. To investigate whether grouping molecular alterations according to the affected signaling pathway could provide a biomarker for TTFields therapy, we scored pathway alterations according to Sanchez et al. [35]. In this analysis, a pathway is considered altered if a case exhibited a critical alteration in at least one pathway‐associated gene. The *TP53* pathway was the most frequently altered (91%, *N* = 58/63), followed by the cell cycle pathway (87%, *N* = 55/63), *RTK/RAS* pathway (80%, *N* = 50/63), and *PI3K/AKT/mTOR* signaling pathway (59%, *N* = 37/63). TMB was characteristically low for pretreatment GBM, IDH wt in our cohort, with a median TMB of 2.4 mutations per mega base.

### 3.4 In TTFields‐treated GBM, IDH wt, homozygous *PTEN* deletion is an adverse prognostic factor

**FIGURE 3:**
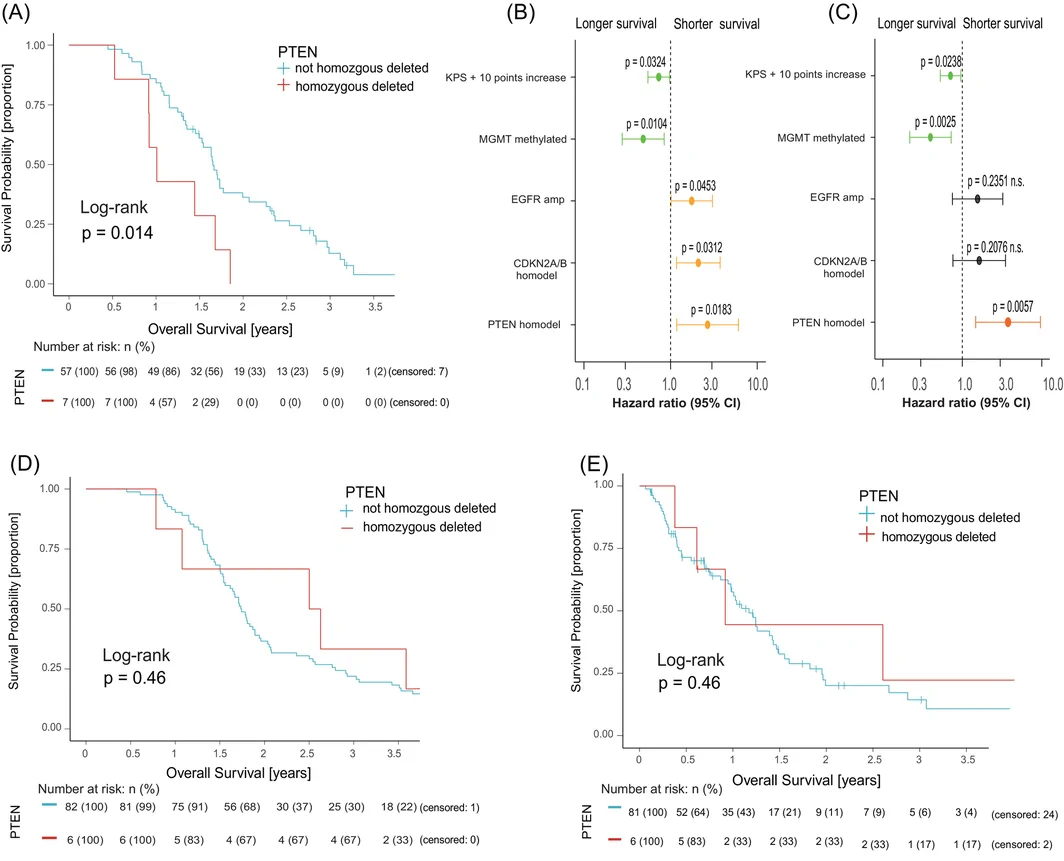
Homozygous *PTEN* deletion is associated with overall survival in TTFields‐treated glioblastoma, IDH wt. (A) Kaplan–Meier analysis of overall survival in TTFields first‐line treated patients with newly diagnosed GBM, IDH‐wt stratified by homozygous *PTEN* deletion status shows decreased outcome for *PTEN* deleted patients. (B) Forest plot of univariate Cox model results. In total 24 factors were investigated by univariate Cox analysis; only the statistically significant factors are shown. (C) Forest plot of the multivariate Cox model. All factors significant in the univariate Cox were included in this analysis. Only KPS, MGMT, and homozygous deletion of *PTEN* remained significant. (D) Kaplan–Meier analysis of overall survival in an independent TTFields‐naïve control cohort with newly diagnosed GBM, IDH wt (Lucas et al.), stratified by *PTEN* deletion status. (E) Kaplan–Meier analysis of overall survival in TTFields‐naïve patients from the TCGA GBM dataset, stratified by *PTEN* deletion status. Log‐rank *p*‐values are shown in each panel. Numbers at risk are indicated below the x‐axes.

Profiling the cohort of 64 molecularly classified GBM, IDH wt revealed 24 molecular alterations detected in at least five patients that allowed further outcome analysis (listed in Table S1). All 24 factors were analyzed for their potential impact on OS. A detailed summary of the results for each individual factor is provided in Table S1 and graphically in Figure S4. In univariate analyses, the following well‐known factors demonstrated a significant protective effect on OS: *MGMT* promoter methylation [Kaplan–Meier estimation: median OS: 741.5 vs. 527 days, *p* = 0.009; univariate Cox model: HR = 0.48 (95% CI: 0.28–0.84), *p* = 0.01] and the clinical factor of preoperative KPS as a continuous variable [univariate Cox model: HR = 0.73 (95% CI: 0.55–0.97), *p* = 0.03]. In addition, the following molecular factors were identified as significant risk factors for poorer OS in univariate analysis: *EGFR* amplification [Kaplan–Meier estimation: median OS: 530 vs. 632 days, *p* = 0.04; univariate Cox model: HR = 1.75 (95% CI: 1.01–3.04), *p* = 0.04] and *CDKN2A/B* homozygous deletion [Kaplan–Meier estimation: median OS: 530 vs. 647 days, *p* = 0.03; univariate Cox model: HR = 1.85 (95% CI: 1.06–3.25), *p* = 0.03] (Figure 3B). Further, homozygous *PTEN* deletion emerged as a particularly strong risk factor associated with shorter survival under TTFields treatment [Kaplan–Meier estimation: median OS: 368 vs. 603 days, *p* = 0.01; univariate Cox model: HR = 2.68 (95% CI: 1.18–6.07), *p* = 0.02] (Figure 3A,B). To determine whether the observed survival association extended to a broader category of putative biallelic *PTEN* inactivation, cases were additionally stratified according to their combined *PTEN* mutation and copy‐number status. Putative biallelic *PTEN* inactivation was present in 26 of 64 tumors, whereas 38 tumors were classified as not harboring biallelic *PTEN* inactivation. In contrast to homozygous *PTEN* deletion alone, putative biallelic *PTEN* inactivation was not significantly associated with OS [Kaplan–Meier estimation: median OS 558 vs. 603 days, log‐rank *p* = 0.61] (Figure S6). To further assess whether genetic *PTEN* alterations translated into loss of *PTEN* protein expression, *PTEN* immunohistochemistry was performed for all cases with available tissue (*n* = 50). Across all investigated tumors, 31 cases showed complete *PTEN* loss (score 0), 14 showed weak/reduced expression (score 1), and 5 retained *PTEN* expression (score 2). Complete *PTEN* loss was observed in all evaluable tumors with *PTEN* homozygous deletion (5/5), and in most tumors with *PTEN* mutation with or without heterozygous deletion (15/17 score 0; 2/17 score 1; 0/17 score 2). In contrast, *PTEN* wildtype tumors with or without heterozygous *PTEN* deletion showed a more heterogeneous staining pattern (11/28 score 0; 12/28 score 1; 5/28 score 2) (Figure S5). However, *PTEN* IHC score itself was not associated with OS. Kaplan–Meier analysis across the three IHC categories showed no significant survival difference (score 0 vs. 1 vs. 2, log‐rank *p* = 0.91). Similarly, complete *PTEN* loss (score 0) was not associated with OS when compared with weak or retained *PTEN* expression (scores 1/2; log‐rank *p* = 0.80) (Figure S5C,D).

All other factors investigated did not reach statistical significance. Kaplan–Meier curves for *CDKN2A/B* homozygous deletion, *EGFR* amplification, *MGMT* promoter status in the TTFields treated cohort and TCGA GBM controls are given in Figure S2A–F. The hazard ratios for all factors significant in univariate Cox analysis are given in Figure 3B.

Two previous studies have reported on molecular biomarkers associated with response to TTFields in glioblastoma [36, 37]. Dono et al. identified *PTEN* mutation as a factor associated with benefit from TTFields treatment [36]. We could not confirm this association, with our univariate analysis demonstrating no statistically significant association [Kaplan–Meier estimation: median OS 632 vs. 596 days, *p* = 0.3; univariate Cox model: HR = 0.76 (95% CI: 0.43–1.35), *p* = 0.35] (Table S1 and Figure S3A). Furthermore, Pandey et al. reported a combination of three molecular markers associated with improved TTFields response (*PIK3CA* wt status, alterations of *NF1* and *EGFR* wt) [37]. In our cohort, *PIK3CA* wt showed no significant survival advantage under TTFields treatment [Kaplan–Meier estimation: median OS 596 vs. 603 days, *p* = 0.7; univariate Cox model: HR = 1.18 (95% CI: 0.50–2.78), *p* = 0.71]. Stratification by *EGFR* status (fusions, splice variants, amplifications, mutations) was in accordance with Pandey et al., with *EGFR* wt status showing a non‐significant trend toward longer OS compared to *EGFR*‐altered tumors [Kaplan–Meier estimation: median OS 632 vs. 530 days, *p* = 0.2; univariate Cox model: HR for *EGFR* wt = 0.69 (95% CI: 0.40–1.21), *p* = 0.19], whereas the presence of *EGFR* alterations was associated with a corresponding trend toward shorter OS [HR for EGFR‐altered = 1.45 (95% CI: 0.83–2.52), *p* = 0.19]. *NF1* alterations (heterozygous deletion, mutation, homozygous deletion) also showed no protective association [Kaplan–Meier estimation: median OS 596 vs. 632 days, *p* = 0.9; univariate Cox model: HR = 1.05 (95% CI: 0.44–2.48), *p* = 0.91]. Survival curves and univariate Cox results are provided in Figure S3.

All clinically and molecularly significant factors identified in the univariate analysis were included in a combined multivariate Cox regression model to assess their impact on OS (Figure 3C). In this analysis, preoperative KPS and *MGMT* promoter methylation emerged as robust protective factors (KPS: HR 0.70, 95% CI 0.52–0.94, *p* = 0.0189, *MGMT*: HR 0.39, 95% CI 0.21–0.72, *p* = 0.0024) underscoring the consistency of our series (Figure 3C). Homozygous *PTEN* deletion also remained significant (HR 3.86, 95% CI: 1.51–9.87, *p* = 0.0049) whereas *EGFR* amplification and *CDKN2A/B* homozygous deletion lost significance (*EGFR*: HR 1.80, 95% CI 0.85–3.84, *p* = 0.1267, *CDKN2A/B*: HR 1.25, 95% CI 0.58–2.70, *p* = 0.5635) (Figure 3C).

In the TTFields treated cohort, the frequency of homozygous *PTEN* deletion was 11% (*N* = 7/64), comparable to 7% (TCGA‐GBM: *N* = 6/87; Lucas et al.: *N* = 6/88) in the non‐TTFields treated control cohorts. Importantly, in the non‐TTFields treated control cohorts, homozygous *PTEN* deletion was not associated with OS (Figure 3D,E).

### 3.5 Molecular analysis reveals that post TTFields GBM, IDH wt do not harbor characteristic molecular alterations

**FIGURE 4:**
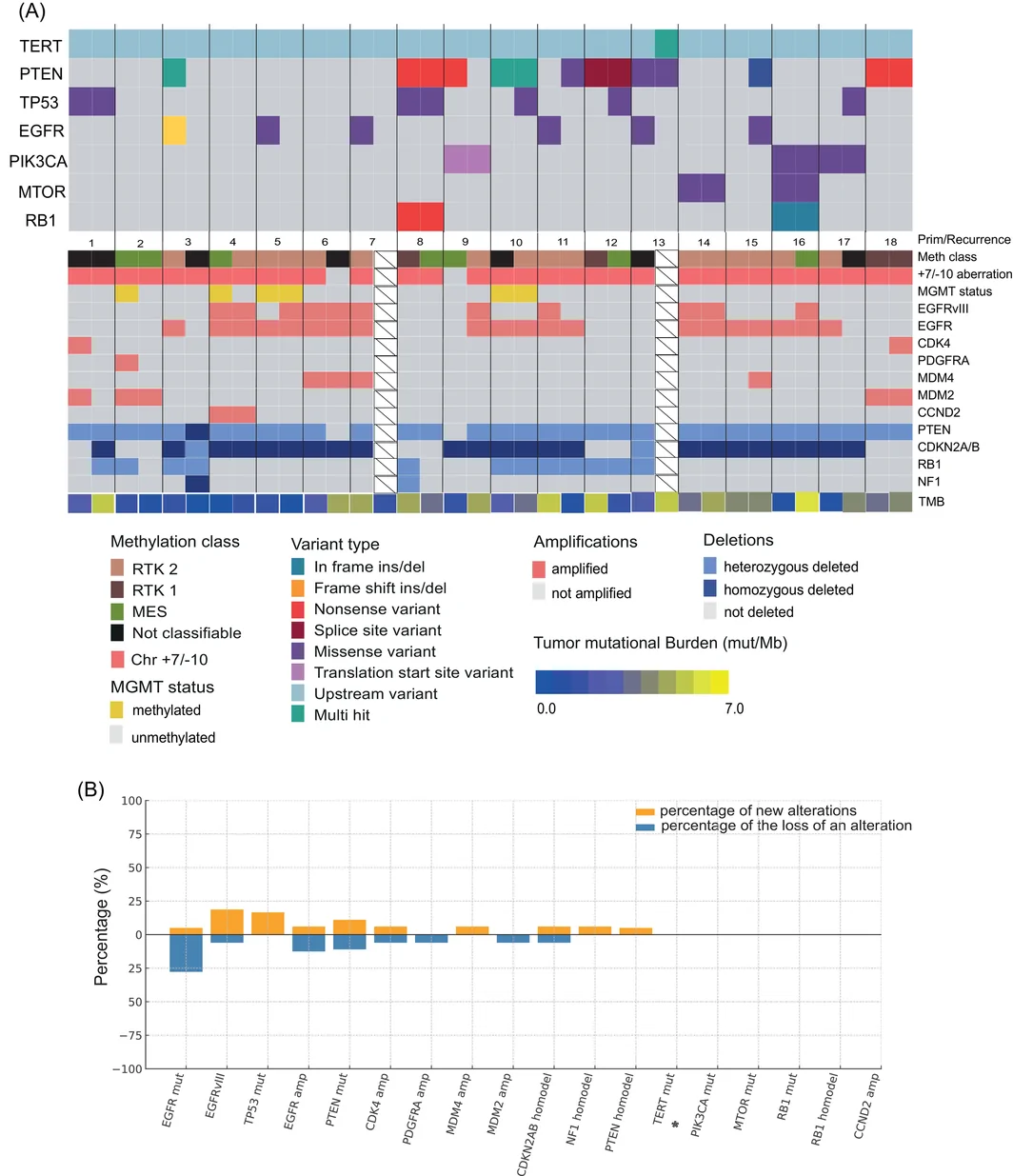
Molecular evolution between initial and recurrent tumors in TTFields first‐line treated glioblastoma, IDH wt. (A) Co‐oncoplot shows frequencies of selected molecular alterations in 18 paired samples at initial diagnosis and at post treatment recurrence. Each patient and the corresponding molecular alterations are represented in two adjacent vertical columns. At the top, driver mutations are shown, with the respective mutation types indicated in the legend. Below, diagnostic markers are depicted, including methylation class (shades of brown, green, and black), chromosome +7/−10 status (light red), *MGMT* promoter methylation (yellow), and copy number changes (light red for amplifications, shades of blue for deletions). Tumor mutational burden (TMB) is shown as a continuous color scale ranging from low (blue) to high (yellow). Grey tiles indicate the absence of an alteration. For two vertical columns, no methylation or copy number data were available because of insufficient tumor cell content. (B) Percentage of cases showing gain or loss of a specific molecular alteration between initial and recurrent tumors, highlighting both newly acquired events (in orange) and loss of alterations (in blue). The Y‐axis indicates the percentage of cases showing a gain (positive) or loss (negative) of a specific molecular alteration. The X‐axis displays the most frequently detected molecular alterations in the 18 tumor pairs of our cohort.

For 18 cases we additionally molecularly investigated post treatment tissue from a second surgery (Figure 4A). Between the two tissue samplings all 18 patients had received radiotherapy and TMZ according to the Stupp protocol as well as TTFields treatment. Overall, only subtle changes of the molecular features before and after treatment were observed (Figure 4A,B). Most notable were a loss of *EGFR* mutation in 5 of 6 cases with *EGFR* mutation and a switch of DNA methylation subclass in 5/18 cases (27%). Similar patterns of molecular evolution have been reported in longitudinally profiled, TTFields‐naïve glioblastoma cohorts. Several studies comparing matched primary and recurrent IDH‐wildtype GBM after standard surgery and radiochemotherapy describe heterogeneous clonal trajectories with both gain and loss of *EGFR* alterations at recurrence rather than a uniformly stable genotype [38, 39]. Regarding DNA methylation class switch, Drexler et al. recently reported a change of the dominant DNA methylation subclass between primary and recurrent surgery in 28.2% of GBM, IDH wt indicating temporal heterogeneity of methylation‐based subclasses without affecting survival. Thus, loss of *EGFR* mutations and methylation subclass transitions in our series are compatible with expected tumor evolution under standard therapy and are not specific to TTFields [40]. Specific molecular alterations that could be attributed to TTFields treatment were not observed, which is consistent with the current understanding that TTFields do not induce genomic mutations [3, 41, 42].

## 4 CONCLUSIONS

In this study, we present a molecularly well‐characterized cohort of newly diagnosed GBM, IDH wt patients who received highly uniform standard‐of‐care treatment concomitant with TTFields. Our results highlight that also in this setting, the classical prognostic markers *MGMT* promoter methylation and preoperative KPS remain robust predictors of overall survival for GBM, IDH wt patients. Importantly, we identified homozygous *PTEN* deletion as an independent adverse prognostic factor specifically in the TTFields treated cohort, whereas this alteration did not show prognostic relevance in non‐TTFields treated control cohorts. In contrast, other molecular alterations previously suggested to be associated with survival in the context of TTFields therapy, including *EGFR*, *NF1*, and *PIK3CA*, did not translate into significant survival differences in our cohort. In a previous small study of 29 patients with recurrent glioblastoma treated with TTFields, Dono et al. reported that *PTEN* mutations were associated with improved post progression survival. In our larger, molecularly confirmed cohort of patients with glioblastoma, IDH wildtype, treated at initial diagnosis, we could not confirm this observation, as *PTEN* mutations were not associated with survival.

Furthermore, our data indicate that homozygous *PTEN* deletion may instead represent a factor associated with worse outcome in TTFields treated patients. *PTEN* deletion status was not investigated by Dono et al., so it remains unclear whether this represents a relevant factor in the recurrent setting.

However, our results raise the question of why certain *PI3K/AKT/mTOR* pathway alterations did not exhibit the same adverse association as homozygous *PTEN* deletion. Neither the composite variable “any alteration” of the *PI3K/AKT/mTOR* pathway nor specific lesions such as *PIK3CA* mutations or *PTEN* point mutations reached statistical significance (Table S1). Similarly, extending the analysis to the broader category of putative biallelic *PTEN* inactivation did not reveal a significant association with OS (log‐rank *p* = 0.61; Figure S6). These findings indicate that distinct alterations within the *PI3K/AKT/mTOR* pathway may differ in their biological consequences and prognostic relevance. In two preclinical studies, homozygous *PTEN* deletion was consistently associated with stronger *AKT* activation, whereas *PIK3CA* alterations—particularly amplifications and hotspot mutations—exhibit more variable and often weaker or phenotypically inconsistent downstream signaling [43, 44]. Less data is available concerning the role of *PTEN* mutations. In glioblastoma, *PTEN* mutations frequently occur together with loss of the second *PTEN* allele as part of the prototypical chromosome 10 loss. However, extending the survival analysis from homozygous *PTEN* deletion to the broader category of putative biallelic *PTEN* inactivation did not reveal a significant association with OS. To further assess whether the genetic *PTEN* alterations observed in our cohort translated into loss of *PTEN* protein expression, we performed *PTEN* immunohistochemistry. As expected, complete *PTEN* loss was observed in all evaluable tumors with homozygous *PTEN* deletion. Tumors harboring *PTEN* mutations with or without heterozygous *PTEN* deletion, mostly occurring in the context of broad chromosome 10 loss, also frequently showed complete or reduced *PTEN* expression. In contrast, tumors with heterozygous *PTEN* deletion alone or without a detectable *PTEN* alteration showed *PTEN* protein loss less frequently, although complete loss was also observed in a subset of these cases. Notably, *PTEN* protein expression assessed by IHC was not associated with OS. Taken together, neither the broader category of putative biallelic *PTEN* inactivation nor immunohistochemically detected *PTEN* protein loss was associated with survival. These findings suggest that the adverse survival association observed in our cohort may be specific to homozygous *PTEN* deletion rather than being explained by biallelic *PTEN* inactivation or *PTEN* protein loss alone. Homozygous *PTEN* deletion may therefore represent a marker of a distinct genomic context associated with poorer outcome in TTFields‐treated patients, although the underlying biological mechanism remains unclear and requires further investigation.

Furthermore, the biological role of *PTEN* in the context of TTFields remains an open question. Both Dono et al. and other preclinical studies implicate *PTEN* in maintaining mitotic spindle assembly and integrity [36, 45]. For example, He et al. demonstrated that *PTEN* orchestrates proper mitotic spindle architecture through regulation of the kinesin motor Eg5, preventing chromosome misalignment and reducing the risk of mitotic catastrophe [46]. As TTFields directly affect the mitotic spindle in cancer cells, *PTEN* status may modulate cellular sensitivity to TTFields. The question of the biological role of *PTEN* in the context of TTFields further leads to the work of Klein‐Goldberg et al. [5]. They observed that TTFields‐induced microtubule disruption activates the *PI3K/AKT/mTOR* signaling pathway, resulting in increased *mTOR* phosphorylation and reduced sensitivity of cancer cells to TTFields. *PTEN* is the principal inhibitor of the *PI3K/AKT/mTOR* pathway [47]. Thus, strong pathway activation caused by homozygous deletion of the key inhibitor *PTEN* may confer pronounced resistance to TTFields in patients carrying homozygous *PTEN* deletion. Notably, *PTEN* loss is not restricted to GBM, IDH wt, but is also observed across several other solid tumors. In non–small cell lung cancer, for example, loss of *PTEN* expression has been reported in roughly 40% of cases and is linked to *PI3K/AKT/mTOR* pathway activation, suggesting that *PTEN* status might also represent a candidate biomarker for future TTFields‐based, biomarker‐driven trials beyond GBM, IDH wt [48].

We additionally analyzed paired GBM, IDH wt samples before and after treatment (including TTFields treatment). This analysis did not reveal evidence of TTFields‐specific recurrent mutations, in line with the current understanding that TTFields exert their antitumoral effects through non‐genotoxic mechanisms. Although preclinical models have repeatedly suggested diverse molecular effects induced by TTFields application, our data do not support an increase in *mTOR* mutations or *CDKN2A* deletions, as had been reported by Robins et al. [42]. The majority of preclinical models have concentrated on RNA transcriptomic and proteomic changes, which cannot be evaluated with the methods used in our study. Furthermore, according to the current state of knowledge, there is no evidence that TTFields cause mutagenic or genotoxic changes leading to DNA mutations [3, 4, 49, 50]. Instead, the changes observed at recurrence were consistent with typical patterns of GBM, IDH wt evolution following radio‐chemotherapy. This, in turn, highlights a clear limitation of this part of our study, as patients received TTFields in addition to standard of care. Such limitations are inherent to analyses of retrospective cohorts.

In conclusion, our results indicate a strong association of homozygous *PTEN* deletion and decreased benefit from TTFields treatment. In the context of clinical decision‐making, exclusion of homozygous *PTEN* deletion may thus be of help for assessing the potential benefits of TTFields treatment. Our data indicate that molecular testing prior TTFields treatment initiation can likely be limited to homozygous *PTEN* deletion analysis. As this can easily be extracted from copy number profiles derived from DNA methylation data that are already routinely generated as part of neuropathological diagnostics in many centers, a prospective evaluation in clinical practice may be warranted and should be relatively straightforward to implement.

## AUTHOR CONTRIBUTIONS

**Jakob Nückles**: Conceptualization; data curation; formal analysis; investigation; visualization; writing – original draft; writing – review & editing. **Claudius Jelgersma**: Investigation; data curation; writing – review & editing. **Christin Siewert**: Formal analysis; data curation; visualization; writing – review & editing. **Brendan Osberg**: Formal analysis; writing – review & editing. **Simone Schmid**: Formal analysis; writing – review & editing. **Arend Koch**: Resources; writing – review & editing. **Eilís Perez**: Writing – review & editing. **Martin Misch**: Writing – review & editing. **Daniel Teichmann**: Formal analysis; writing – review & editing. **Anna‐Gila Karbe**: Writing – review & editing. **Susanne Ukrow**: Resources; writing – review & editing. **Patrick N. Harter**: Resources; writing – review & editing. **Ruth M. Stassart**: Resources; writing – review & editing. **Peter Vajkoczy**: Writing – review & editing. **Julia Onken**: Conceptualization; supervision; funding acquisition; writing – review & editing. **David Capper**: Conceptualization; project administration; supervision; funding acquisition; methodology; data curation; writing – review & editing.

## FUNDING INFORMATION

The molecular analyses in this study were funded by Novocure®. The funder had no role in study design, data collection and analysis, decision to publish, or preparation of the manuscript.

## CONFLICT OF INTEREST STATEMENT

D.C. declares a patent on DNA methylation‐based methods for molecular classification of tumor species and holds shares of Heidelberg Epignostix GmbH, Heidelberg, Germany. The other authors have no conflicts of interest to declare.

## ETHICS STATEMENT

This study was approved by the ethics committee of the Charité‐Universitätsmedizin, Berlin, Germany (proposal number: EA4/154/22). This study was conducted in accordance with the local legislation and institutional requirements. Informed consent was obtained from all individual participants included in the study.

## Supporting information


**Figure S1.** Shown are CNV plots calculated from DNA methylation data using the v12.8 classifier version. Gains/amplifications are represented as positive deviations from the baseline in green, and losses as negative deviations in red. The x‐axis displays chromosomes 1–22, while the y‐axis shows the corresponding logarithmic scale. (A) This case demonstrates a gain of more than 50% of chromosome arm 7, whereas more than half of chromosome 10 is lost. The classifier output (upper right) does not reach a sufficient class score of 0.84 but instead shows 0.74. Together with additional NGS data, such as the detection of a *TERT* promoter mutation, this case was classified as GBM, IDH wt. The CNV plot further illustrates how heterozygous deletions were classified. For GBM cases with chromosome 10 loss, the level of chromosome 10 loss was used as a reference measurement. *CDKN2A/B*, *PTEN*, and *RB1* are considered heterozygously deleted in this example, as their copy number losses do not fall below the mean level of chromosome 10 loss. In contrast, *NF1* was classified as balanced, since its copy number level is approximately at baseline. (B) This example CNV plot illustrates the ideal case of a very high class score of 0.99 for glioblastoma, IDH wt and a confident subclass score of 0.99 for receptor tyrosine kinase 2 (*RTK2*). It provides a representative example of the definition of high‐level amplification (in this example *EGFR*), characterized by a focal upward shift of the baseline (blue) accompanied by a series of distinct green dots covering the gene locus, typically with a log2 value greater than 0.6. A homozygous deletion of *CDKN2A/B* is also present. (C) Further illustrates a CNV plot with the characteristic +7/−10 chromosomal aberration, reflecting trisomy of chromosome 7 and monosomy of chromosome 10. Of particular importance is the depiction of homozygous losses of *CDKN2A/B* and *PTEN*. A homozygous gene loss was defined as present when the deviation from the baseline (blue) was at least twice the difference between the chromosome 10 loss and the baseline.

**Figure S2.** Kaplan–Meier analysis of overall survival (OS) in patients with newly diagnosed glioblastoma, IDH‐wildtype (GBM, IDH wt). Log‐rank *p*‐values are shown in each panel. Numbers at risk are indicated below the x‐axes. The TTFields‐treated cohort was stratified by (A) homozygous *CDKN2A/B* deletion, (C) *EGFR* amplification, and (E) *MGMT* promoter methylation status. For comparison, the TCGA control cohort was stratified by the corresponding alterations: (B) homozygous *CDKN2A/B* deletion, (D) *EGFR* amplification, and (F) *MGMT* promoter methylation status.

**Figure S3.** Kaplan–Meier and Cox model survival analysis of TTFields‐treated cohort stratified by molecular alterations reported by Dono et al. and Pandey et al. Log‐rank *p*‐values are shown in each panel. Numbers at risk are indicated below the x‐axes. (A) *PTEN* mutation status (Dono et al.). (B) *PIK3CA* mutation status (Pandey et al.). (C) *NF1* alteration status (Pandey et al.). (D) *EGFR* alteration status (Pandey et al.). (E) Forest plot of univariate Cox proportional hazards models for the respective molecular alterations.

**Figure S4.** Forest plot of univariate Cox proportional hazards analyses for overall survival in 64 patients with GBM, IDH wt treated with TTFields. Each marker represents the hazard ratio (HR) for the comparison indicated on the y‐axis, with horizontal lines showing the corresponding 95% confidence interval on a logarithmic scale. The vertical dashed line indicates HR = 1. Green markers and bars denote factors associated with a reduced hazard (HR < 1), orange markers and bars denote factors associated with an increased hazard (HR > 1).

**Figure S5.** *PTEN* immunohistochemistry and association with *PTEN* genotype and overall survival. (A) Representative *PTEN* immunohistochemistry images illustrating the three‐tier scoring system used in this study: retained *PTEN* expression (score 2), weak or reduced *PTEN* expression (score 1), and complete loss of *PTEN* expression in tumor cells (score 0). (B) Distribution of *PTEN* IHC scores across grouped *PTEN* genotypes in evaluable cases. Bars represent the relative proportion of cases with complete *PTEN* loss (score 0), weak/reduced expression (score 1), or retained expression (score 2); n indicates the number of evaluable cases per group. (C) Kaplan–Meier analysis of overall survival stratified by *PTEN* IHC score showed no significant survival difference between score 0, score 1, and score 2 tumors (log‐rank *p* = 0.91). (D) Kaplan–Meier analysis comparing complete *PTEN* loss (score 0) with weak or retained *PTEN* expression (scores 1/2) likewise showed no significant association with overall survival (log‐rank *p* = 0.80). Numbers at risk are shown below the Kaplan–Meier curves.

**Figure S6.** Kaplan–Meier analysis of overall survival according to putative biallelic *PTEN* inactivation. No significant survival difference was observed between tumors with and without putative biallelic *PTEN* inactivation (log‐rank *p* = 0.61). Numbers at risk are shown below the plot.

**Table S1.** Molecular alterations analyzed for association with overall survival in the TTFields‐treated GBM, IDH‐wildtype cohort.

**Table S2.** Univariate Cox regression analysis of clinical variables associated with overall survival.

## Data Availability

Raw idat files are made publicly available under Array express number E‐MTAB‐16255. Raw Sequencing data (FASTQ files) from DNA Sequencing is publicly available under European Genome‐phenome Archive (EGA) study accession EGAS50000001469.
