# Supplementary material for: PTEN homozygous deletion is a negative prognostic factor in tumor treating fields‐treated glioblastoma, IDH wildtype patients
Source: Brain Pathol. 2026 Sep 10:e70143. Online ahead of print. doi: 10.1111/bpa.70143 (PMC13561735; doi:10.1111/bpa.70143)
Supplement: Supplementary file 1 [file BPA-9999-e70143-s001.pdf]

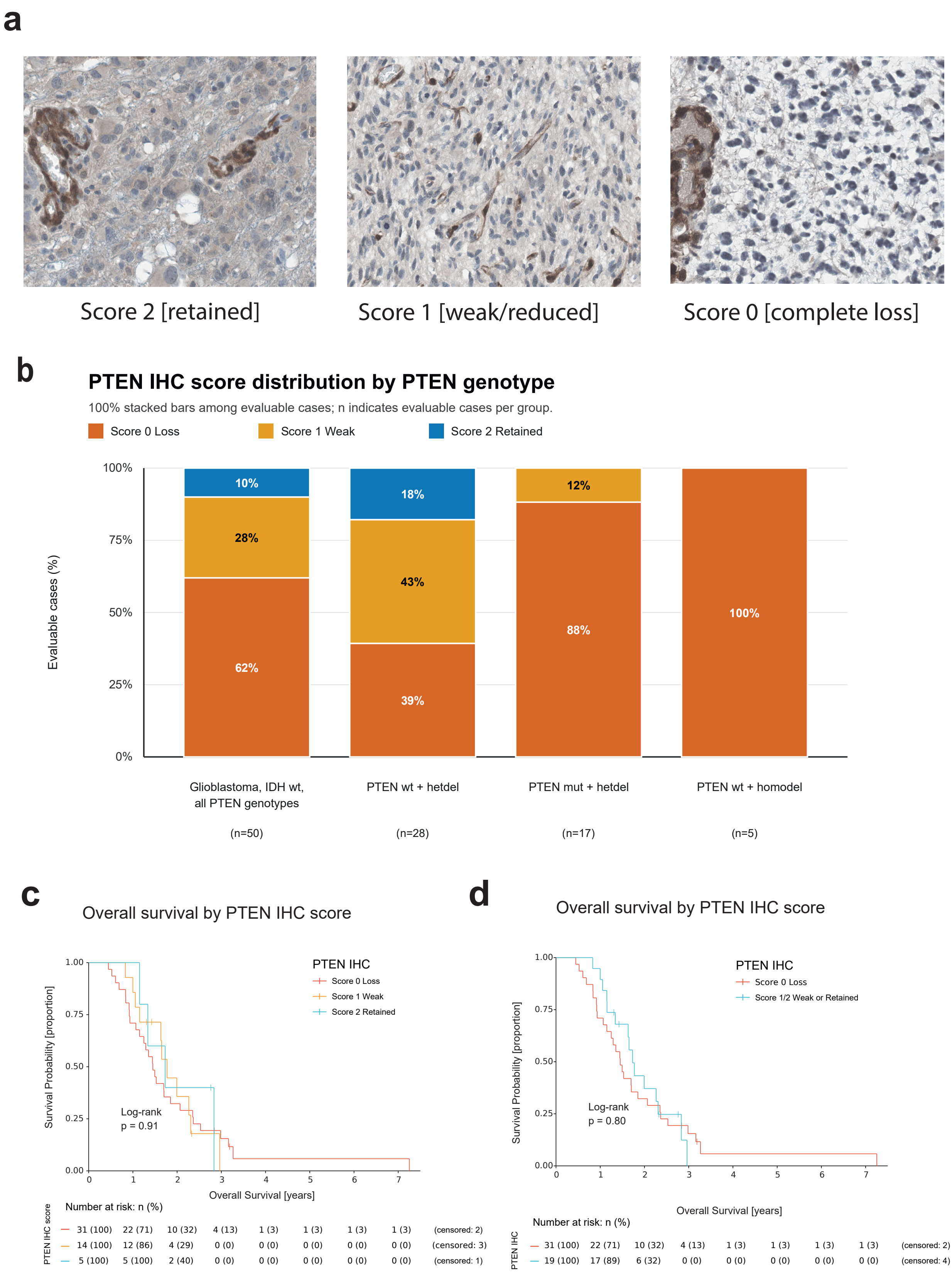
a
Score 2 [retained]
Score 1 [weak/reduced]
Score 0 [complete loss]
b
PTEN IHC score distribution by PTEN genotype
100% stacked bars among evaluable cases; n indicates evaluable cases per group.
Score 0 Loss
Score 1 Weak
Score 2 Retained
Evaluable cases (%)
100%
75%
50%
25%
0%
10%
28%
62%
18%
43%
39%
12%
88%
100%
Glioblastoma, IDH wt, all PTEN genotypes
PTEN wt + hetdel
PTEN mut + hetdel
PTEN wt + homodel
(n=50)
(n=28)
(n=17)
(n=5)
c
Overall survival by PTEN IHC score
PTEN IHC
Score 0 Loss
Score 1 Weak
Score 2 Retained
Survival Probability [proportion]
1.00
0.75
0.50
0.25
0.00
Log-rank
p = 0.91
0 1 2 3 4 5 6 7
Overall Survival [years]
Number at risk: n (%)
PTEN IHC score
31 (100) 22 (71) 10 (32) 4 (13) 1 (3) 1 (3) 1 (3) 1 (3) (censored: 2)
14 (100) 12 (86) 4 (29) 0 (0) 0 (0) 0 (0) 0 (0) 0 (0) (censored: 3)
5 (100) 5 (100) 2 (40) 0 (0) 0 (0) 0 (0) 0 (0) 0 (0) (censored: 1)
d
Overall survival by PTEN IHC score
PTEN IHC
Score 0 Loss
Score 1/2 Weak or Retained
Survival Probability [proportion]
1.00
0.75
0.50
0.25
0.00
Log-rank
p = 0.80
0 1 2 3 4 5 6 7
Overall Survival [years]
Number at risk: n (%)
PTEN IHC
31 (100) 22 (71) 10 (32) 4 (13) 1 (3) 1 (3) 1 (3) 1 (3) (censored: 2)
19 (100) 17 (89) 6 (32) 0 (0) 0 (0) 0 (0) 0 (0) 0 (0) (censored: 4)
