# Supplementary material for: PTEN homozygous deletion is a negative prognostic factor in tumor treating fields‐treated glioblastoma, IDH wildtype patients
Source: Brain Pathol. 2026 Sep 10:e70143. Online ahead of print. doi: 10.1111/bpa.70143 (PMC13561735; doi:10.1111/bpa.70143)
Supplement: Supplementary file 2 [file BPA-9999-e70143-s002.pdf]

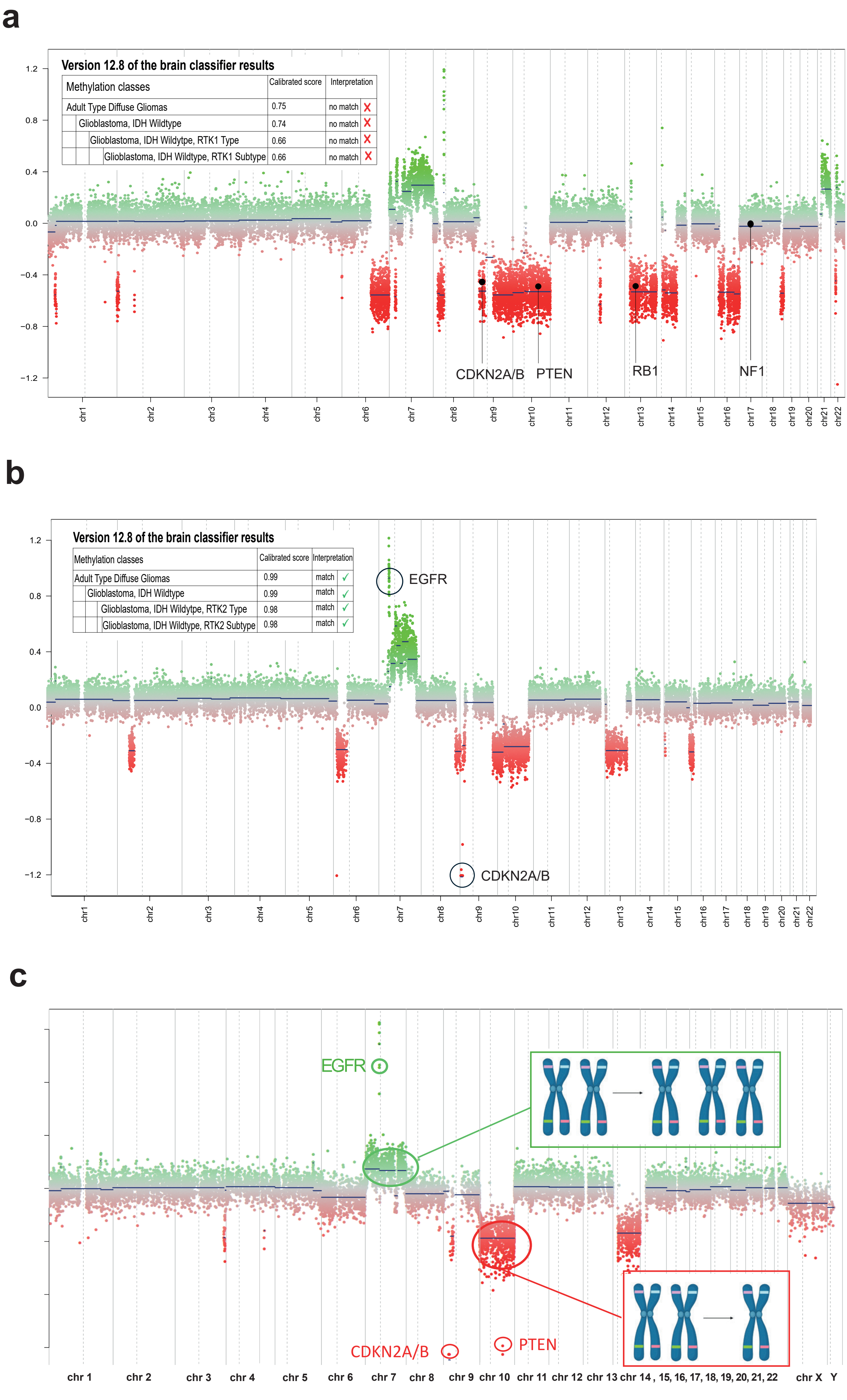
a
Version 12.8 of the brain classifier results
Methylation classes
Calibrated score
Interpretation
Adult Type Diffuse Gliomas 0.75 no match
Glioblastoma, IDH Wildtype 0.74 no match
Glioblastoma, IDH Wildtype, RTK1 Type 0.66 no match
Glioblastoma, IDH Wildtype, RTK1 Subtype 0.66 no match
1.2
0.8
0.4
0.0
−0.4
−0.8
−1.2
CDKN2A/B
PTEN
RB1
NF1
chr1 chr2 chr3 chr4 chr5 chr6 chr7 chr8 chr9 chr10 chr11 chr12 chr13 chr14 chr15 chr16 chr17 chr18 chr19 chr20 chr21 chr22
b
Version 12.8 of the brain classifier results
Methylation classes
Calibrated score
Interpretation
Adult Type Diffuse Gliomas 0.99 match
Glioblastoma, IDH Wildtype 0.99 match
Glioblastoma, IDH Wildtype, RTK2 Type 0.98 match
Glioblastoma, IDH Wildtype, RTK2 Subtype 0.98 match
1.2
0.8
0.4
0.0
−0.4
−0.8
−1.2
EGFR
CDKN2A/B
chr1 chr2 chr3 chr4 chr5 chr6 chr7 chr8 chr9 chr10 chr11 chr12 chr13 chr14 chr15 chr16 chr17 chr18 chr19 chr20 chr21 chr22
c
EGFR
CDKN2A/B
PTEN
chr 1 chr 2 chr 3 chr 4 chr 5 chr 6 chr 7 chr 8 chr 9 chr 10 chr 11 chr 12 chr 13 chr 14 , 15, 16, 17, 18, 19, 20, 21, 22 chr X Y
