# Supplementary material for: PTEN homozygous deletion is a negative prognostic factor in tumor treating fields‐treated glioblastoma, IDH wildtype patients
Source: Brain Pathol. 2026 Sep 10:e70143. Online ahead of print. doi: 10.1111/bpa.70143 (PMC13561735; doi:10.1111/bpa.70143)
Supplement: Supplementary file 3 [file BPA-9999-e70143-s004.pdf]

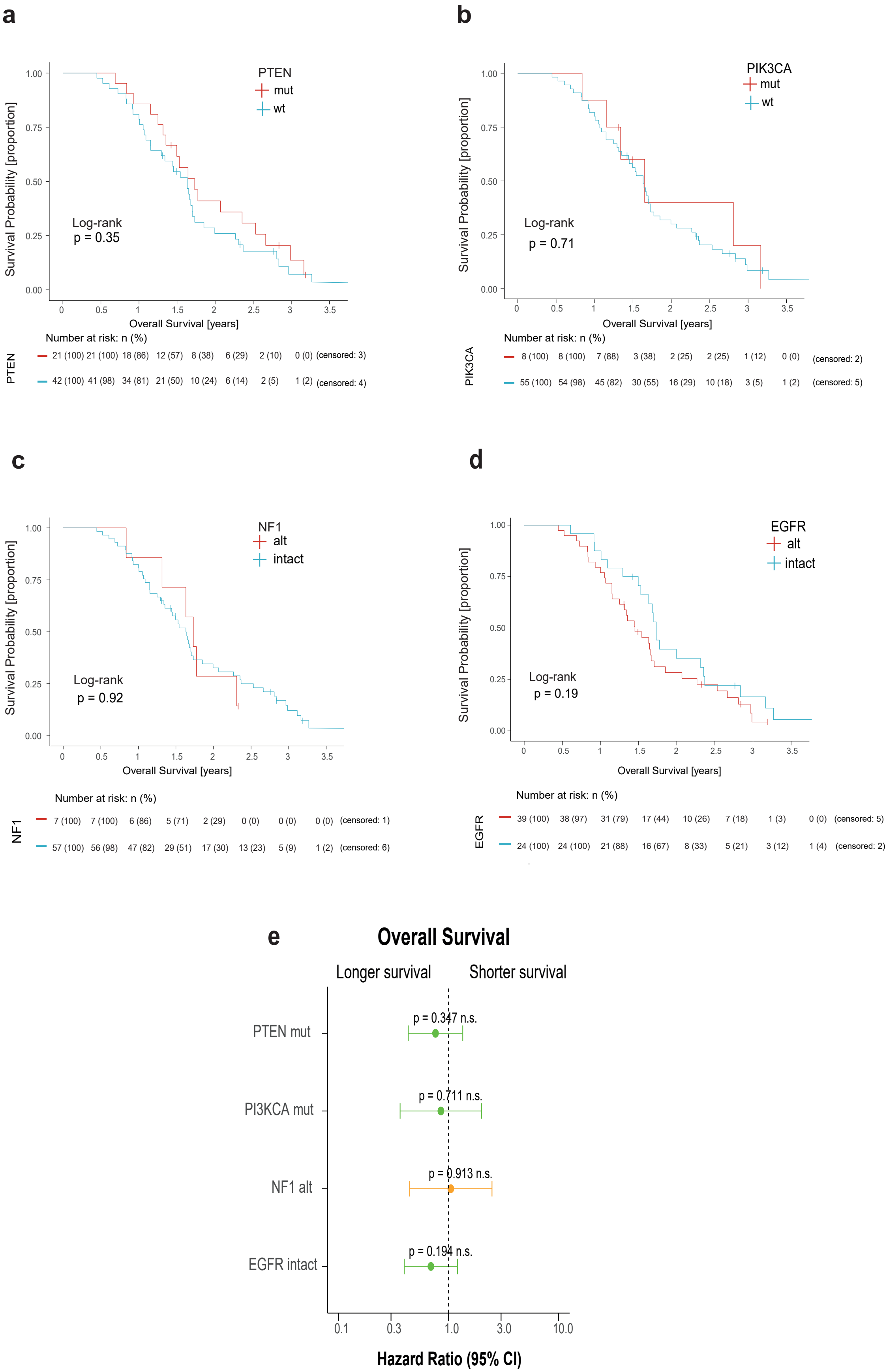
a
PTEN
mut
wt
Survival Probability [proportion]
Log-rank
p = 0.35
Overall Survival [years]
Number at risk: n (%)
PTEN
21 (100) 21 (100) 18 (86) 12 (57) 8 (38) 6 (29) 2 (10) 0 (0) (censored: 3)
42 (100) 41 (98) 34 (81) 21 (50) 10 (24) 6 (14) 2 (5) 1 (2) (censored: 4)
b
PIK3CA
mut
wt
Survival Probability [proportion]
Log-rank
p = 0.71
Overall Survival [years]
Number at risk: n (%)
PIK3CA
8 (100) 8 (100) 7 (88) 3 (38) 2 (25) 2 (25) 1 (12) 0 (0) (censored: 2)
55 (100) 54 (98) 45 (82) 30 (55) 16 (29) 10 (18) 3 (5) 1 (2) (censored: 5)
c
NF1
alt
intact
Survival Probability [proportion]
Log-rank
p = 0.92
Overall Survival [years]
Number at risk: n (%)
NF1
7 (100) 7 (100) 6 (86) 5 (71) 2 (29) 0 (0) 0 (0) 0 (0) (censored: 1)
57 (100) 56 (98) 47 (82) 29 (51) 17 (30) 13 (23) 5 (9) 1 (2) (censored: 6)
d
EGFR
alt
intact
Survival Probability [proportion]
Log-rank
p = 0.19
Overall Survival [years]
Number at risk: n (%)
EGFR
39 (100) 38 (97) 31 (79) 17 (44) 10 (26) 7 (18) 1 (3) 0 (0) (censored: 5)
24 (100) 24 (100) 21 (88) 16 (67) 8 (33) 5 (21) 3 (12) 1 (4) (censored: 2)
e
Overall Survival
Longer survival
Shorter survival
PTEN mut
p = 0.347 n.s.
PI3KCA mut
p = 0.711 n.s.
NF1 alt
p = 0.913 n.s.
EGFR intact
p = 0.194 n.s.
Hazard Ratio (95% CI)
