# Supplementary material for: PTEN homozygous deletion is a negative prognostic factor in tumor treating fields‐treated glioblastoma, IDH wildtype patients
Source: Brain Pathol. 2026 Sep 10:e70143. Online ahead of print. doi: 10.1111/bpa.70143 (PMC13561735; doi:10.1111/bpa.70143)
Supplement: Supplementary file 4 [file BPA-9999-e70143-s005.pdf]

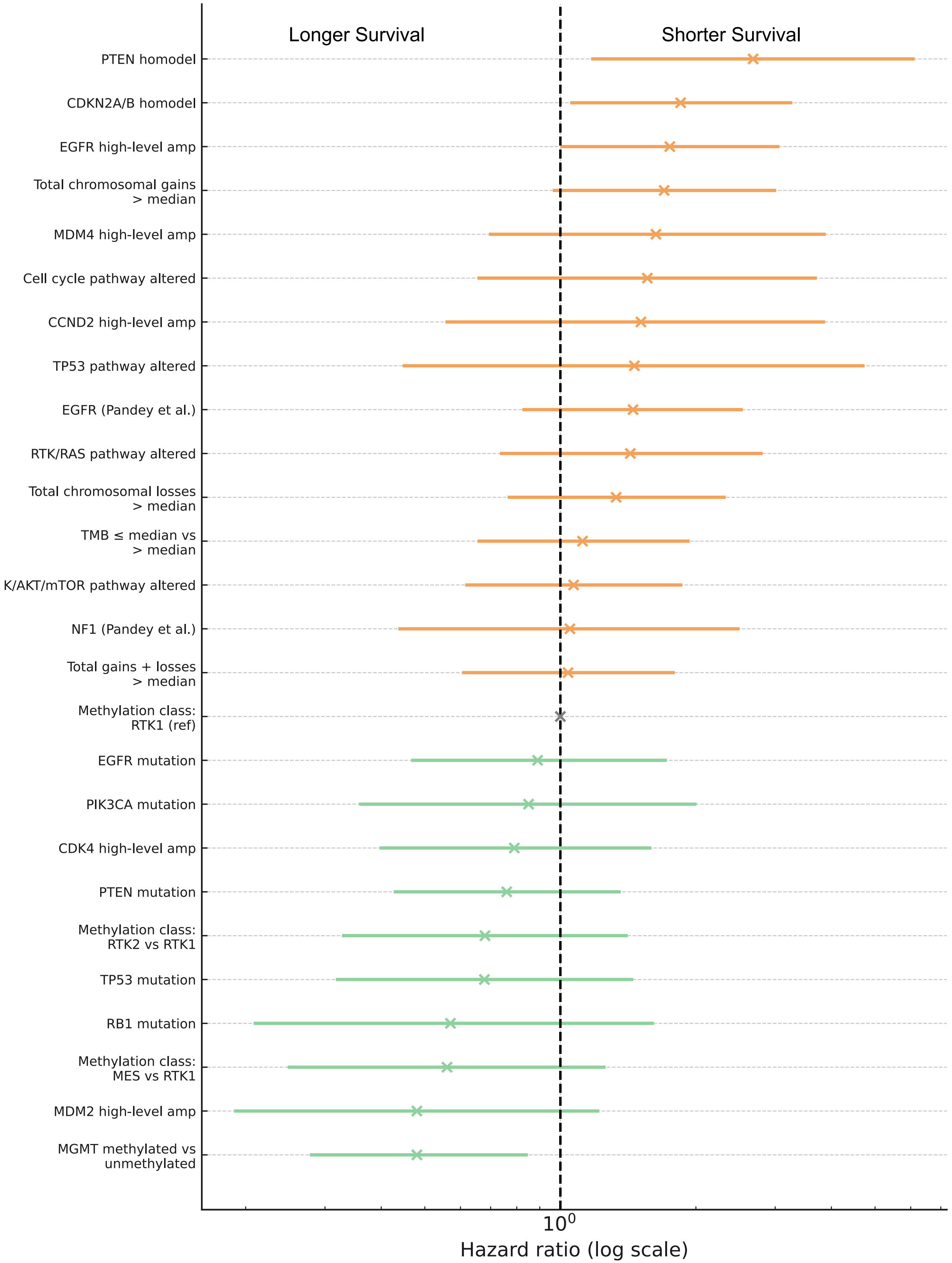
Longer Survival
Shorter Survival
PTEN homodel
CDKN2A/B homodel
EGFR high-level amp
Total chromosomal gains > median
MDM4 high-level amp
Cell cycle pathway altered
CCND2 high-level amp
TP53 pathway altered
EGFR (Pandey et al.)
RTK/RAS pathway altered
Total chromosomal losses > median
TMB ≤ median vs > median
K/AKT/mTOR pathway altered
NF1 (Pandey et al.)
Total gains + losses > median
Methylation class: RTK1 (ref)
EGFR mutation
PIK3CA mutation
CDK4 high-level amp
PTEN mutation
Methylation class: RTK2 vs RTK1
TP53 mutation
RB1 mutation
Methylation class: MES vs RTK1
MDM2 high-level amp
MGMT methylated vs unmethylated
10^0
Hazard ratio (log scale)
