# Supplementary material for: PTEN homozygous deletion is a negative prognostic factor in tumor treating fields‐treated glioblastoma, IDH wildtype patients
Source: Brain Pathol. 2026 Sep 10:e70143. Online ahead of print. doi: 10.1111/bpa.70143 (PMC13561735; doi:10.1111/bpa.70143)
Supplement: Supplementary file 5 [file BPA-9999-e70143-s007.pdf]

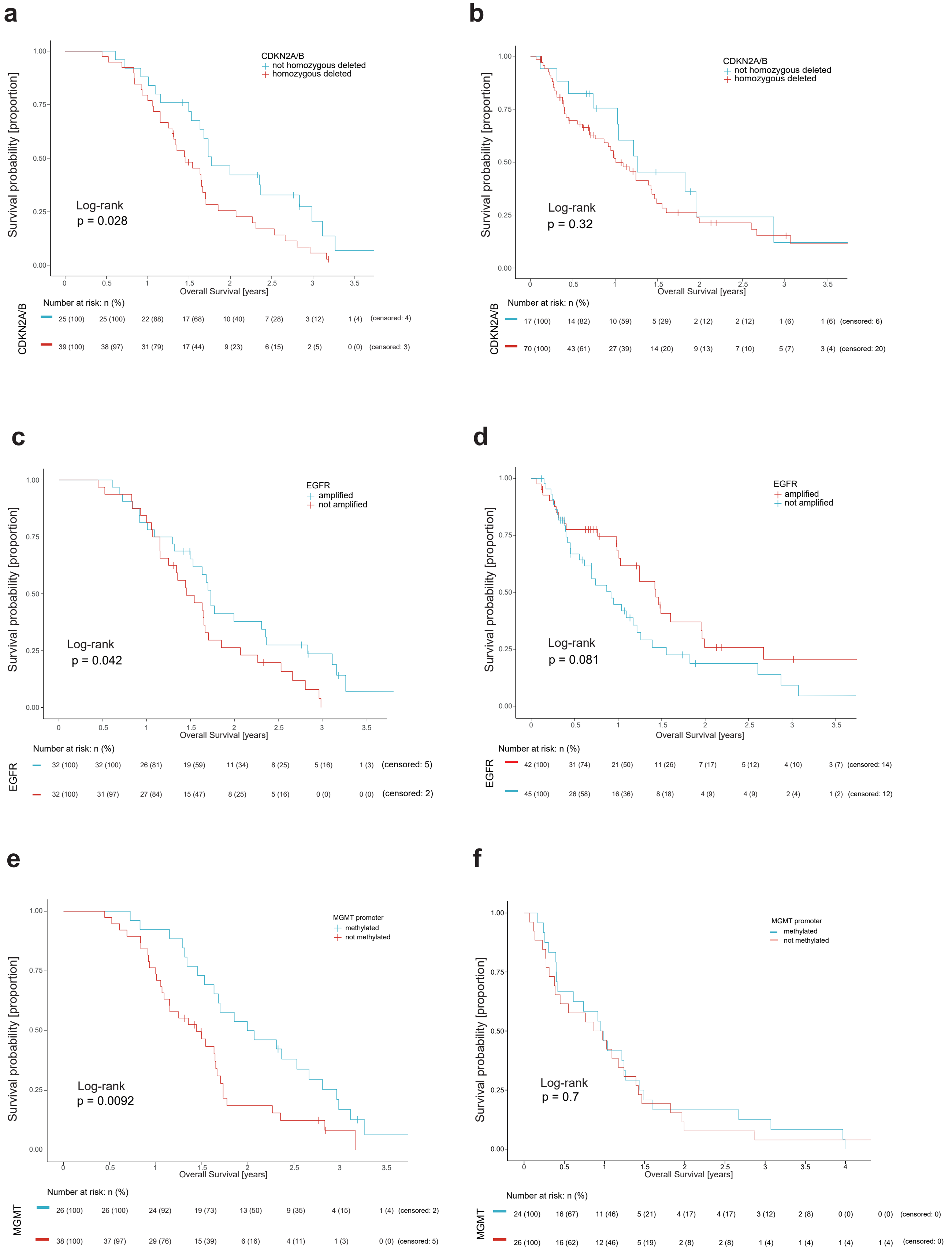

a
CDKN2A/B
not homozygous deleted
homozygous deleted
Log-rank
p = 0.028
Survival probability [proportion]
Overall Survival [years]
Number at risk: n (%)
CDKN2A/B
25 (100) 25 (100) 22 (88) 17 (68) 10 (40) 7 (28) 3 (12) 1 (4) (censored: 4)
39 (100) 38 (97) 31 (79) 17 (44) 9 (23) 6 (15) 2 (5) 0 (0) (censored: 3)
b
CDKN2A/B
not homozygous deleted
homozygous deleted
Log-rank
p = 0.32
Survival probability [proportion]
Overall Survival [years]
Number at risk: n (%)
CDKN2A/B
17 (100) 14 (82) 10 (59) 5 (29) 2 (12) 2 (12) 1 (6) 1 (6) (censored: 6)
70 (100) 43 (61) 27 (39) 14 (20) 9 (13) 7 (10) 5 (7) 3 (4) (censored: 20)
c
EGFR
amplified
not amplified
Log-rank
p = 0.042
Survival probability [proportion]
Overall Survival [years]
Number at risk: n (%)
EGFR
32 (100) 32 (100) 26 (81) 19 (59) 11 (34) 8 (25) 5 (16) 1 (3) (censored: 5)
32 (100) 31 (97) 27 (84) 15 (47) 8 (25) 5 (16) 0 (0) 0 (0) (censored: 2)
d
EGFR
amplified
not amplified
Log-rank
p = 0.081
Survival probability [proportion]
Overall Survival [years]
Number at risk: n (%)
EGFR
42 (100) 31 (74) 21 (50) 11 (26) 7 (17) 5 (12) 4 (10) 3 (7) (censored: 14)
45 (100) 26 (58) 16 (36) 8 (18) 4 (9) 4 (9) 2 (4) 1 (2) (censored: 12)
e
MGMT promoter
methylated
not methylated
Log-rank
p = 0.0092
Survival probability [proportion]
Overall Survival [years]
Number at risk: n (%)
MGMT
26 (100) 26 (100) 24 (92) 19 (73) 13 (50) 9 (35) 4 (15) 1 (4) (censored: 2)
38 (100) 37 (97) 29 (76) 15 (39) 6 (16) 4 (11) 1 (3) 0 (0) (censored: 5)
f
MGMT promoter
methylated
not methylated
Log-rank
p = 0.7
Survival probability [proportion]
Overall Survival [years]
Number at risk: n (%)
MGMT
24 (100) 16 (67) 11 (46) 5 (21) 4 (17) 4 (17) 3 (12) 2 (8) 0 (0) 0 (0) (censored: 0)
26 (100) 16 (62) 12 (46) 5 (19) 2 (8) 2 (8) 1 (4) 1 (4) 1 (4) 1 (4) (censored: 0)
