# Supplementary material for: PTEN homozygous deletion is a negative prognostic factor in tumor treating fields‐treated glioblastoma, IDH wildtype patients
Source: Brain Pathol. 2026 Sep 10:e70143. Online ahead of print. doi: 10.1111/bpa.70143 (PMC13561735; doi:10.1111/bpa.70143)
Supplement: Supplementary file 6 [file BPA-9999-e70143-s008.pdf]

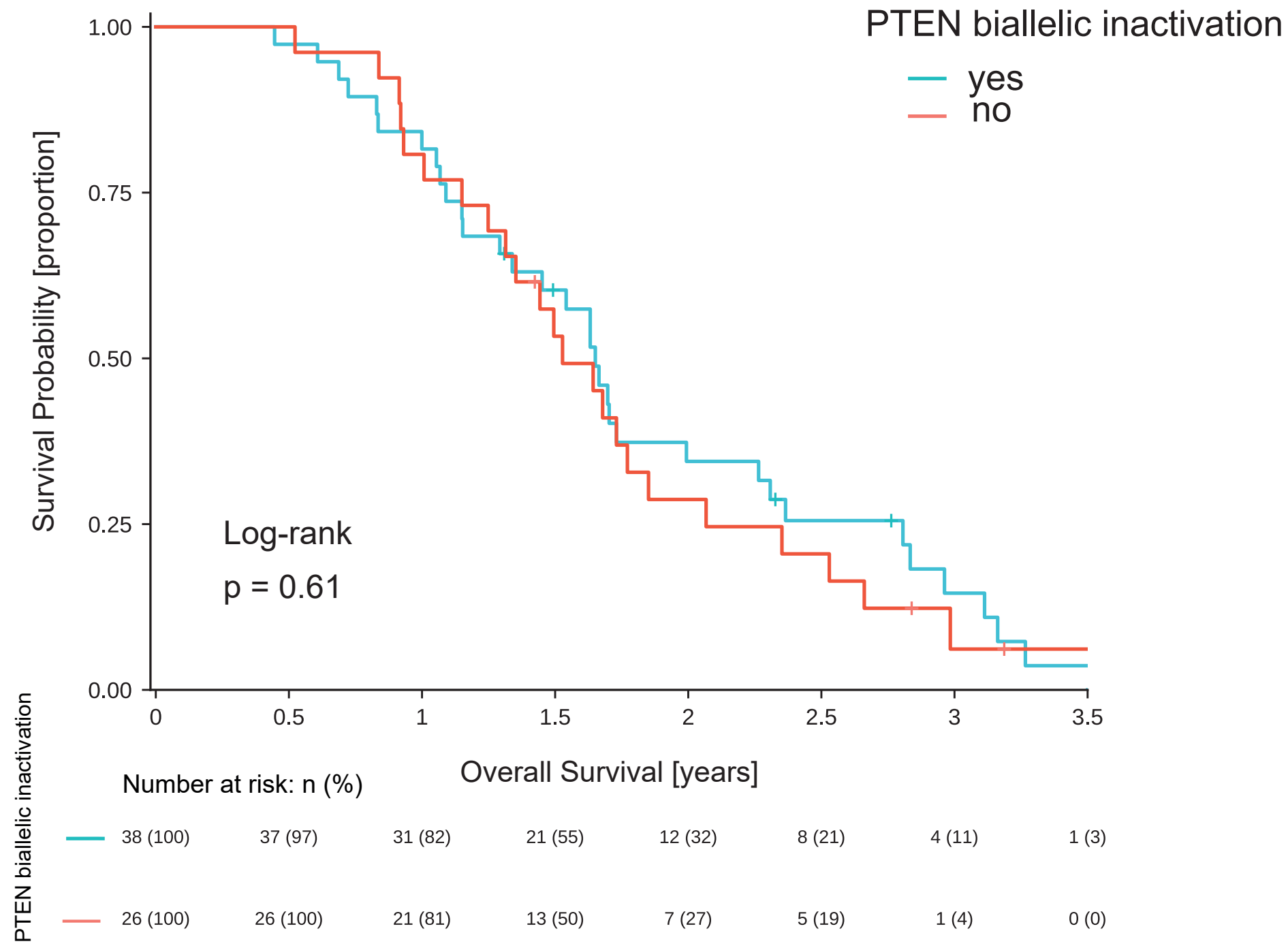
PTEN biallelic inactivation
yes
no
Survival Probability [proportion]
1.00
0.75
0.50
0.25
0.00
Log-rank
p = 0.61
0
0.5
1
1.5
2
2.5
3
3.5
Overall Survival [years]
Number at risk: n (%)
PTEN biallelic inactivation
38 (100) 37 (97) 31 (82) 21 (55) 12 (32) 8 (21) 4 (11) 1 (3)
26 (100) 26 (100) 21 (81) 13 (50) 7 (27) 5 (19) 1 (4) 0 (0)
